# Routine Lateral Level V Dissection May Not Be Necessary for Papillary Thyroid Microcarcinoma With Lateral Lymph Node Metastasis: A Retrospective Study of 252 Cases

**DOI:** 10.3389/fendo.2019.00558

**Published:** 2019-08-20

**Authors:** Shuai Xue, Peisong Wang, Qiang Zhang, Yue Yin, Liang Guo, Ming Wang, Meishan Jin, Guang Chen

**Affiliations:** ^1^Department of Thyroid Surgery, The First Hospital of Jilin University, Changchun, China; ^2^Department of Pathology, The First Hospital of Jilin University, Changchun, China

**Keywords:** level V dissection, papillary thyroid microcarcinoma, lateral lymph node metastasis, lateral lymph node dissection, recurrence

## Abstract

**Background:** Lateral lymph node metastasis (LLNM) is associated with distant metastasis, locoregional recurrence and cancer-specific mortality, although the prevalence of LLNM among patients with papillary thyroid microcarcinoma (PTMC) is relatively low. The potential benefits and risks of routine lateral level V dissection (LVD) for PTMC with LLNM have not been previously investigated.

**Methods:** A total of 6,880 consecutive PTMC patients who underwent initial surgery at the First Hospital of Jilin University from January 2009 to July 2017 were retrospectively analyzed. A total of 252 N1b PTMC patients were enrolled in our study.

**Results:** The overall and occult metastasis rates in level V lymph nodes were 21.4 and 6.4%, respectively. Patients with N1b PTMC who received LVD did not show a significantly lower disease-free survival (DFS) than that of patients who did not receive LVD [hazard ratio = 1.11 (CI 0.38–3.21); *p* = 0.85]. Meanwhile, LVD simultaneously increased the hospital stay and cost (*p* = 0.03; 0.02). Multivariate logistic regression analysis revealed that 3-level simultaneous metastasis in the lateral neck was an independent risk factor for level V metastasis [odds ratio = 8.6 (CI 1.42–51.72); *p* = 0.02].

**Conclusions:** Because of the low metastasis rate in level V lymph nodes, the lack of benefit for recurrence, the longer hospital stay and the higher cost associated with LVD, N1b PTMC patients without clinical level V metastasis may not need to undergo routine dissection. Prophylactic LVD may be recommended only for patients with N1b PTMC with 3-level simultaneous metastasis.

## Introduction

The global incidence of papillary thyroid microcarcinoma (PTMC), which is defined as papillary thyroid carcinoma (PTC) measuring ≤1 cm in greatest dimension, has increased appreciably (1, 2). The vast majority of PTMCs are indolent with a good prognosis, but a small group of PTMC patients may have locoregional recurrence, which is still a major concern for physicians (3). Regional lymph node metastasis (LNM), especially in the lateral neck, is associated with higher locoregional recurrence and poorer prognosis for PTMC patients (4, 5). Because of the high incidence of multilevel lateral lymph node metastasis (LLNM) and persistent/recurrent disease in the lateral neck, modified radical neck dissection (MRND) is recommended by the American Thyroid Association (ATA) for PTMC patients with clinically metastatic lymph nodes in the lateral compartment (6).

MRND is performed to remove all the lateral lymph nodes and fibro-fatty tissues, including those throughout levels II–V, with the preservation of the internal jugular vein, spinal accessory nerve, and sternocleidomastoid muscle (7, 8). For PTC larger than 1 cm, clinical benefits and risks of routine level V dissection (LVD) have been reported even though the results are still controversial (9–11). Although lateral neck compartment involvement is less common in PTMC patients, whether the LLNM pattern of PTMC is the same as that of PTC with >1 cm tumors is still debatable (12–14). Moreover, PTMC patients with LLNM exhibit more frequent multifocality and skip metastasis and less central lymph node metastasis (CLNM) than do PTC patients with tumors larger than 1 cm (14, 15). Whether level V lymph nodes should be routinely dissected in PTMC patients with LLNM remains unknown because of the lack of evidence.

The aim of this study was to evaluate the potential benefits and risks of routine lateral LVD for PTMC with LLNM. In this study, we would such as to demonstrate several clinical concerns: (1) whether routine lateral LVD decreases locoregional recurrence; (2) whether routine lateral LVD increases LVD-related complications; (3) what clinicopathological features predict level V metastasis can help physicians differentiate N1b PTMC patients with level V metastasis and further guide treatment strategies.

## Materials and Methods

### Patient Selection

The Institutional Review Board of the First Hospital of Jilin University approved this study, and written informed consent was waived due to the retrospective nature of the study. A total of 6,880 consecutive PTMC patients who underwent initial surgery at the First Hospital of Jilin University from January 2009 to July 2017 were retrospectively analyzed. The inclusion criteria for patient selection were as follows: patient information was found in the hospital database; patients underwent total thyroidectomy with central lymph node dissection (CLND) with therapeutic lateral lymph node dissection (LLND) as the initial surgery; and the postoperative pathological diagnosis was PTMC with LLNM. The exclusion criteria were as follows: patients aged <18 years (4 cases); those with persistent disease (1 case); those with a history of neck radiotherapy (0 case); and those with a history of previous thyroid surgery (8 cases). Finally, 252 N1b PTMC patients were enrolled in our study.

### Diagnosis and Treatment

The majority of PTMC patients were diagnosed by routine ultrasound (US) examination, which was conducted by a trained radiologist (Y Yin) and preoperatively by surgeons (S Xue, PS Wang, Q Zhang) to evaluate a thyroid tumor and neck lymph nodes. US features of malignant lymph nodes were microcalcifications, cystic aspect, peripheral vascularity, hyperechogenicity, and rounded shape. If the largest diameter of cervical lymph node was larger than 0.8 cm and presented with one or more US malignant features, FNA for suspicious lymph nodes was recommended for patients. Computed tomography (CT) scans were only performed for PTMC patients with FNA-proven LNM for the surgical plan. CLND was performed as previously described; (16) LLND was only performed for patients with clinical N1, which was diagnosed using US, CT, and FNA. Because there is a lack of consensus regarding routine lateral LVD, patients chose MRND (level II to V) or selective lateral neck dissection (SLND) (level II to IV) according to their preference. Radioiodine ablation (RAI) and TSH-suppressive hormonal therapy were recommended for postoperative patients according to established guidelines as previously reported (17).

### Histopathological Examination

Histological specimens were examined and independently reviewed by two pathologists (L Guo and M Wang). Histopathological characteristics, including the diameter of all tumors, bilaterality, number of tumor foci, extrathyroidal extension (ETE), histological variant, presence of Hashimoto thyroiditis (HT), and LNM, were recorded. Few discordant cases were discussed with the experienced pathologist (MS Jin). Occult lymph node metastases are defined as microscopic metastases of tumor deposits which are initially undetectable by US, CT, or FNA preoperatively and subsequently identified by pathology postoperatively.

### Follow-Up and Recurrence

All patients were followed up with physical examinations, serum unstimulated thyroglobulin (Tg) levels, Tg antibody detection, and US at 6- to 12-months intervals. Patients who received RAI were recommended to receive diagnostic iodine-131 whole-body scans. When recurrence was suspected, patients underwent FNA with or without the measurement of washout Tg levels and thyroid CT. Recurrence was defined as the presence of a tumor or metastatic lymph node in a patient who was considered clinically free of disease at least 6 months after the initial surgery.

### Statistical Analysis

Nominal variables are described as frequencies, and proportions and continuous variables are presented as the means and standard deviations (SDs). To identify differences between groups for specific variables, Pearson's chi-square tests were used for nominal variables and the Mann-Whitney *U* test for continuous variables. Disease-free survival (DFS) curves were drawn using Kaplan-Meier methods and statistically analyzed using the log-rank test. For multivariate logistic regression analysis, continuous variables were turned into nominal variables using cutoffs that were calculated using receiver operating characteristic curve (ROC) analysis. *P* < 0.05 were considered statistically significant (2-sided). SPSS version 22 software (SPSS Inc., Chicago, IL) was used for all statistical analyses.

## Results

### Baseline Characteristics

The baseline clinicopathological characteristics of the 252 N1b PTMC patients are summarized in Table 1. One hundred sixty-six (65.9%) patients were females, and the average age of all patients was 38.7 years. The majority of patients (78.9%) had multifocal disease. Only six (2.4%) patients had gross ETE, while microscopic ETE was identified among 219 (86.9%) of the N1b PTMC patients. Overall, 104 (41.3%) patients received left LLND, 138 (54.8%) received right LLND, and 10 (3.9%) received bilateral LLND. Skip metastasis was found among 19 (7.5%) patients. One hundred seventy-two patients (68.3%) suffered lateral multilevel metastasis. The average follow-up duration was 55.69 ± 23.37 months. Recurrence was detected in 20 (8.0%) of the 252 N1b PTMC patients.

**Table 1:** Clinicopathological characteristics of N1b PTMC patients.

**Variables**	***N* = 252 (%)**
**Sex**	
Female	166 (65.9)
Male	86 (34.1)
**Age, years**	38.7 ± 10.2
<55	235 (93.3)
≥55	17 (6.7)
**Bilateral**	
Yes	117 (46.4)
No	135 (53.6)
**Location of tumor**	
Solitary tumor	71 (28.1)
Upper third	36 (14.3)
Middle third	20 (7.9)
Lower third	15 (6.0)
Multifocal tumor	181 (78.9)
In both lobes	117 (46.4)
In one lobe	64 (25.4)
**ETE**	
No	27 (10.7)
Microscopic	219 (86.9)
Gross	6 (2.4)
**Histology variants**	
Conventional	248 (98.4)
Follicular	4 (1.6)
**LTD (cm)**	0.73 ± 0.2
**HT**	
Yes	166 (65.9)
No	86 (34.1)
**LLND side**	
Left	104 (41.3)
Right	138 (54.8)
Both	10 (3.9)
**Regional LNM**	
Central	233 (92.5)
Lateral	252 (100)
**Extent of LLNM**	
Single-level	80 (31.7)
Multi-level	172 (68.3)
**RAI ablation**	
Yes	208 (82.5)
No	44 (17.5)
**Recurrence**	
Yes	20 (8.0)
No	232 (92.0)
**Follow up (months)**	55.69 ± 23.37

*Categorical variables are presented as number (%, percentage). Continuous variables are presented as the average ± standard deviation. PTMC, papillary thyroid microcarcinoma; ETE, extrathyroidal extension; LTD, largest tumor diameter; HT, Hashimoto's thyroiditis; LLND, lateral lymph neck dissection; LNM, lymph node metastasis; LLNM, lateral lymph node metastasis; RAI, radioactive iodine*.

### Distribution of LLNM Among the 252 N1b Patients

Among the 252 N1b patients, the metastatic rates for levels II, III, and IV were 56.5, 50.4, and 72.9%, respectively (Table 2). For 56 patients who underwent LVD, only 12 (21.4) patients had LNM in level V lymph nodes. Of the clinically negative lateral neck levels without suspicious lymph nodes diagnosed by US, CT, or FNA preoperatively, the occult metastatic rates were 28.35% (45/159), 26.1% (46/176), 34.9% (38/109), and 6.4% (3/47) in lateral neck levels II, III, IV, and V, respectively (Table 2).

**Table 2:** Distribution of LLNM in 252 N1b patients.

**Level**	**Overall metastasis**			**Occult metastasis**		
	**Metastatic levels**	**Dissected levels**	**Percentage (%)**	**Metastatic levels**	**Dissected levels**	**Percentage (%)**
II	148	262	56.5	45	159	28.3
III	132	262	50.4	46	176	26.1
IV	191	262	72.9	38	109	34.9
V	12	56	21.4	3	47	6.4

*LLNM, lateral lymph node metastasis*.

### Recurrence and Surgical Complications According to Lateral LVD

**Figure 1:**
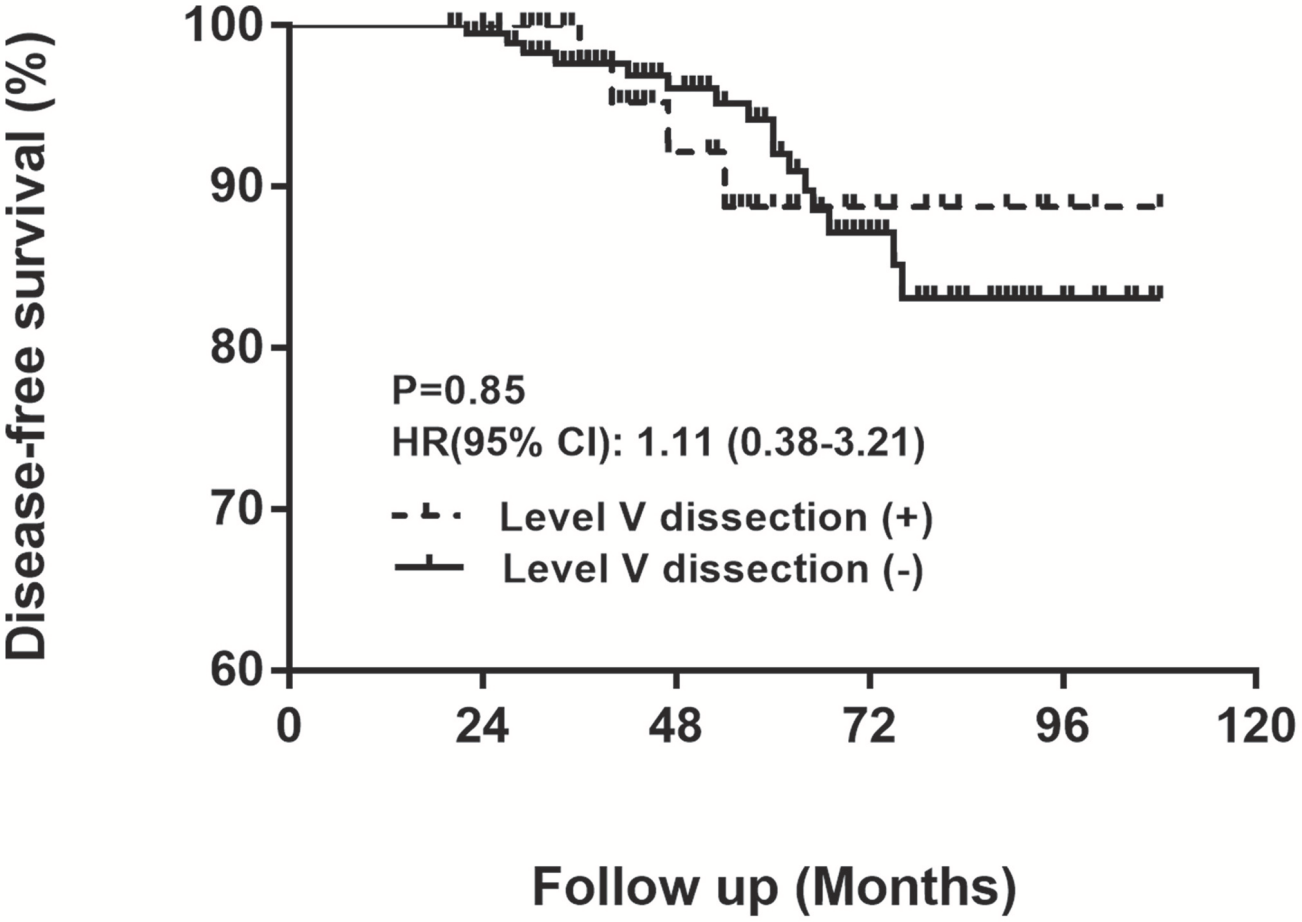
Comparison of disease-free survival rate according to LVD.

To compare the recurrence rates and surgical complications between N1b PTMC patients with LVD and those without LVD, baseline clinicopathological characteristics, including sex, age, BMI, bilaterality, multifocality, ETE, histological variants, largest tumor diameter (LTD), HT, LLND side, CLNM, extent of LLNM, RAI ablation, surgeon, and follow-up duration, were compared between these two groups, as shown in Table 3. None of these confounders, which may affect recurrence or surgical complications, were significantly different between these two groups. Meanwhile, 7.1% (4/56) of N1b PTMC tumors in patients who received LVD and 8.2% (16/196) of N1b PTMC tumors in patients who did not receive LVD exhibited recurrence (*p* = 1.00). Figure 1 illustrates the comparison of DFS rate according to LVD and shows that patients with LVD did not exhibit significantly lower DFS rates [hazard ratio = 1.11 [CI 0.38–3.21]; *p* = 0.85].

**Table 3:** Baseline clinicopathological features comparison of N1b PTMC patients with and without LVD.

**Variables**	**Level V dissection (+) No. (%), *N* = 56**	**Level V dissection (–) No. (%), *N* = 196**	***P*-value**
**Sex**			
Female	33 (59.0)	133 (67.9)	
Male	23 (41.0)	63 (32.1)	0.21
**Age, years**	37.25 ± 9.0	39.11 ± 10.56	0.36
**BMI (m**^**2**^**/kg)**	23.78 ± 11.3	24.44 ± 15.7	0.42
**Bilateral**			
Yes	29 (51.8)	88 (44.9)	
No	27 (48.2)	108 (55.1)	0.36
**Location of tumor**			0.55^*^
Solitary tumor	14	57	0.72^#^
Upper third	6	30	
Middle third	4	16	
Lower third	4	11	
Multifocal tumor	42	139	0.50^&^
In both lobes	29	88	
In one lobe	13	51	
**ETE**			
No	6 (10.7)	21 (10.7)	
Microscopic	46 (78.6)	171 (87.2)	
Gross	4 (7.1)	4 (2.1)	0.21
**Histology variants**			
Conventional	54 (96.4)	195 (99.5)	
Follicular	2 (3.6)	1 (0.5)	0.13
**LTD (cm)**	0.74 ± 0.21	0.72 ± 0.20	0.54
**HT**			
Yes	20 (35.7)	66 (33.7)	
No	36 (64.3)	130 (66.3)	0.78
**MLND side**			
Left	25 (44.6)	79 (40.3)	
Right	31 (55.4)	107 (54.6)	
Both	0 (0)	10 (5.1)	0.07
**CLNM**	4.61 ± 4.53	3.77 ± 3.54	0.33
**LLNM**	9.11 ± 8.21	9.98 ± 8.07	0.97
**Extent of LLNM**			
Single-level	25 (44.6)	66 (33.7)	
Multi-level	31 (55.4)	130 (66.3)	0.15
**LLLND (cm)**	1.64 ± 0.63	1.59 ± 0.78	0.78
**RAI ablation**			
Yes	45 (80.4)	163 (83.2)	
No	11 (19.6)	33 (16.8)	0.63
**Operator**			
Prof. A	23 (41.1)	58 (29.6)	
Prof. B	13 (23.2)	56 (28.6)	
Prof. C	11 (19.6)	48 (24.5)	
Prof. D	9 (16.1)	34 (17.3)	0.44
**Recurrence**			
Yes	4 (7.1)	16 (8.2)	
No	52 (92.9)	180 (91.8)	1
**Follow up (months)**	56 ± 23	56 ± 24	0.77

PTMC, papillary thyroid microcarcinoma; ETE, extrathyroidal extension; LTD, largest tumor diameter; HT, Hashimoto's thyroiditis; CLNM, central lymph node metastasis; LLNM, lateral lymph node metastasis; LLLND, largest lateral lymph node diameter; RAI, radioactive ablation; Prof, professor; ^*^, Solitary vs. Multifocality; ^#^, For solitary tumors, upper third vs. middle third vs. lower third; ^&^, For multifocal tumors, both lobes vs. single lobe.

Furthermore, hospitalization and surgical complications, such as length of hospital stay, total cost, surgical time, blood loss during surgery, drainage volume, hemorrhage, incision infection, lymphorrhagia, and shoulder syndrome, were compared among N1b PTMC patients according to whether or not they received LVD. LVD simultaneously increased hospital stay and cost (*p* = 0.03; 0.02). However, surgical complications related to neck dissection were low among both groups, and none of the complications were remarkably different between the groups, as shown in Table 4.

**Table 4:** Hospitalization and complications in N1b PTMC patients according to level V dissection.

**Variables**	**Level V dissection (+) No. (%), *N* = 56**	**Level V dissection (–) No. (%), *N* = 196**	***P***
**Hospital stay (days)**	12.24 ± 2.56	9.81 ± 2.33	**0.03**
**Hospital cost (dollars)**	3512 ± 278	2910 ± 301	**0.02**
**Surgery time (min)**	176.33 ± 68.11	172.69 ± 55.31	0.91
**Blood loss during surgery (ml)**	40.54 ± 17.35	42.11 ± 20.07	0.87
Drainage volume (ml)^*^	185.42 ± 78.31	169.17 ± 49.87	0.06
**Hemorrhage**			
Yes	0 (0)	0 (0)	
No	56 (100)	196 (100)	1.00
**Incision infection**			
Yes	0 (0)	1 (0.5)	
No	56 (100)	195 (99.5)	1.00
**Lymphorrhagia**			
Yes	2 (3.6)	6 (3.1)	
No	54 (96.4)	190 (96.9)	1.00
**Shoulder syndrome**			
Yes	1 (1.8)	1 (0.5)	
No	55 (98.2)	195 (99.5)	0.92

PTMC, papillary thyroid microcarcinoma;

* *Drainage information were available in 36 of 56 PTMC patients with level V dissection and 112 of 196 patients without level V dissection. Bold P value: statistically significant*.

### Univariate and Multivariate Analysis of Risk Factors for Level V Metastasis

To investigate risk factors for level V metastasis in PTMC patients by logistic regression analysis, continuous variables such as age, LTD and CLNM were converted to nominal variables using cutoffs that were calculated using ROC analysis (Supplementary Table 1). LLNM was defined according to surgical pathology postoperatively. As Table 5 shows, univariate and multivariate analyses revealed that 3-level simultaneous metastasis in the lateral neck was an independent risk factor for level V metastasis [*p* = 0.04 for univariate analysis; OR = 8.6 [CI 1.42–51.72] and *p* = 0.02 for multivariate analysis].

**Table 5:** Univariate and multivariate analysis of clinicopathological characteristics for level V metastasis in PTMC patients.

**Variables**	**Level V metastasis**		**Univariate analysis**	**Multivariate analysis**	
	**Present, *n* = 12**	**Absent, *n* = 44**	***P***	**OR (95% CI)**	***P***
**Sex**					
Female	7 (58.3)	26 (59)			
Male	5 (41.7)	18 (41)	1.00	NA	NA
**Age**					
≤32 year	2 (16.7)	17 (38.7)			
>32 year	10 (83.3)	27 (61.3)	0.28	NA	NA
**Bilateral**					
Yes	6 (50.0)	23 (52.2)		NA	
No	6 (50.0)	21 (47.8)	0.89		NA
**ETE**					
No	2 (16.7)	4 (9.0)			
Microscopic	8 (66.6)	38 (86.4)			
Gross	2 (16.7)	2 (4.6)	0.29	NA	NA
**Histology variants**					
Conventional	11 (90.9)	43 (97.7)			
Follicular	1 (9.1)	1 (2.3)	0.9	NA	NA
**LTD (cm)**					
≤0.6	2 (16.7)	15 (34.1)			
>0.6	10 (83.3)	29 (65.9)	0.42	NA	NA
**Multifocality**					
Yes	7 (58.3)	35 (79.6)			
No	5 (41.7)	9 (20.4)	0.26	NA	NA
**CLNM**					
≤1	5 (41.7)	9 (20.5)			
>1	7 (58.3)	35 (79.5)	0.26	NA	NA
**HT**					
Yes	5 (41.7)	15 (34.0)			
No	7 (58.3)	29 (66.0)	0.15	NA	NA
**Simultaneous metastasis**					
1- level	2 (16.7)	23 (59.1)		1 (reference)	NA
2- level	4 (33.3)	13 (29.6)		3.5 (0.57–22.03)	0.18
3- level	6 (50.0)	8 (11.3)	**0.04**	8.6 (1.42–51.72)	**0.02**

*PTMC, papillary thyroid microcarcinoma; OR, odd ratio; ETE, extrathyroidal extension; LTD, largest tumor diameter; CLNM, central lymph node metastasis; HT, Hashimoto's thyroiditis. Bold P value: statistically significant*.

## Discussion

To our knowledge, this is the first study to investigate the potential benefits and risks of lateral LVD in N1b PTMC patients. Mounting evidence has been published to prove that LLNM is associated with distant metastasis, locoregional recurrence and cancer-specific mortality, although the prevalence of LLNM among patients with PTMC is relatively low, ranging from 1.1 to 9.4% (4, 5, 12, 13, 18–21). Multiple guidelines recommend MRND from level II to V for N1b patients for either PTC or PTMC (6, 22). However, our study demonstrated that the occult metastatic rate of N1b PTMC in level V was only 6.4%. After adjusting for all potential confounders for recurrence, patients with N1b PTMC who received LVD did not show a higher DFS rate than that of patients who did not undergo LVD. Moreover, patients who received LVD spent more time in the hospital and money on the hospitalization costs even if they did not suffer significantly more surgical complications. In agreement with other studies, we also identified that 3-level metastasis was an independent risk factor for level V metastasis in patients with N1b PTMC (9).

Compared with levels II, III, and IV, the overall incidence of LLNM in level V was much lower, ranging from 4.5 to 33.9%, as previously reported (14, 15, 23, 24). In our study, the overall and occult metastasis rates of level V lymph nodes were 21.4 and 6.4%, which were also the lowest among all lateral lymph node levels. Moreover, single-level metastasis in level V was not observed in our study as well as research by Liu et al. (23) In another study that summarized 215 N1b PTMC patients, only 1 patient (0.5%, 1/215) presented with single-level metastasis in level V (24). In addition, skip metastasis was found in 19 (7.5%) patients in our study, which is in agreement with the results in other publications (13, 15, 19, 25). However, in our study as well as in other N1b PTMC cohorts, skip metastasis to single-level metastasis in level V was not reported. The evidence summarized above suggests that lateral level V lymph nodes may be considered as the subsequent station following level II, III, or IV when lymphatic metastasis occurs from thyroid carcinoma, although much more convincing proof is needed in the future.

These “berry picking” procedures are not recommended for N1b thyroid cancer patients because of the obviously higher locoregional recurrence (26). The main reason for MRND is to reduce recurrence, prevent reoperation and avoid complications related to resurgery. However, as shown in our study, performing LVD in N1b PTMC patients did not affect the recurrence rate, which may be attributed to the relatively lower overall and occult metastasis rates in lateral level V lymph nodes. Despite the potential benefits of LVD, surgical-related complications have also been observed. Complications, including shoulder syndrome (shoulder dysfunction or shoulder pain for more than 6 months), did not occur more frequently in the LVD group. Kim SK et al. reported a higher shoulder syndrome rate (9.1, 24/263) in PTC patients who received LVD (9). We explain these differences as follows. (1) In our department, lateral neck dissections were performed only by professors. Lymph node dissection by a high-volume surgeon may lead to lower surgical complications. (2) Surgeons recommended routine intraoperative neuromonitoring for all N1b patients. Neuromonitoring stimulation to the accessory nerve for shoulder movement also helps surgeons identify nerves. (3) Generally, level V is the last compartment to be dissected in our department. Therefore, the accessory nerve has always been identified during level II, III, and IV dissection before LVD. Additionally, LVD increased the patients' length of hospital stay and associated cost according to our study. The average drainage volume was 185.42 ± 78.31 in LVD group, which was higher than patients without LVD although the difference was not statistically significant. Probably, delayed removal of drainage were the reasons for the longer hospital stay and associated cost in our study.

Previously, the accuracy and cost-effectiveness of diagnosing level V metastasis by imaging have been poor. We aimed to identify risk factors of level V metastasis and help clinicians decide what type of patients may need prophylactic LVD. In our study, 3-level simultaneous metastasis was a risk factor for level V metastasis, which was also reported in PTC patients previously (9, 27, 28). Compared with level V metastasis in 1-level metastasis (2/25, 8%), it was more frequent in 2-level metastasis (4/17, 23.5%) although the different was not statistically significant by multivariate analysis. The more levels of metastasis in lateral neck (level II, III, and IV), the more likely level V had metastatic lymph nodes. It may be because multilevel metastasis represents more aggressive tumor. Since this is the first study in which the predictive factors for level V metastasis in N1b PTMC patients were investigated, future validation studies for these results are warranted.

Several limitations in the study must be noted. First, it was a retrospective study at a tertiary medical center. Potential selection bias may exist, and further prospective studies are required. Second, the average follow-up time was 4.6 years (56 months), which may be shorter than the recurrence time for PTMC. Some patients with shorter follow-up times may exhibit recurrence in the future. The recurrence rate without statistical difference between PTMC patients with LVD and without LVD may attribute to short follow-up times. The result should be interpreted with caution and a cohort with a longer follow-up is needed. Third, because of the low incidence of LLNM in PTMC patients, the relatively small number of patients may also generate some bias in our study. Also, the small number of patients limits the probability of propensity score matching to eliminate potential cofounders when we compare DFS between PTMC patients with LVD and without LVD. Some unaware and unmeasured confounders may influence the strength of the result although many factors have been taken into consideration for baseline comparison, as shown in Table 3. Fourth, N1b PTMCs with 3-level simultaneous metastasis were more frequent for level V metastasis. However, 3-level simultaneous metastasis (including clinical and microscopic) was defined according to surgical pathology postoperatively. Surgical extent may not be planned preoperatively based on US or during surgery based on frozen section. Further study which will investigate preoperative risk factors of level V metastasis is needed. In addition, these clinical care strategies, like hospitalization time, use of drains and cost, for PTMC patients with MLND were different from those in other countries. The potential discrepancy may limit the generalizability of these findings on other hospitals. Finally, the majority of recurrent patients were followed up by telephone, and the patients could not provide the exact information about the recurrent lymph node location. Therefore, we could not calculate the level V recurrence rate, which may also be important for this study. However, the number of level V recurrent cases were more likely small according to pretty low occult metastasis rate in level V (3/47, 6.4%) and other studies previously published (9, 27, 28).

## Conclusion

Because of the low metastasis rate in level V lymph nodes, the lack of benefit for recurrence, the longer hospital stays and the higher cost associated with LVD, level V lymph nodes may not need to be routinely dissected in N1b PTMC patients without clinical level V metastasis. Prophylactic LVD may be recommended only for N1b PTMC with 3-level simultaneous metastasis.

## Data Availability

The datasets generated for this study are available on request to the corresponding author.

## Synopsis

LVD did not show a DFS benefit for N1b PTMC patients, which might be attributed to relatively lower overall and occult metastasis rates. Meanwhile, LVD increased hospital stay and cost. Prophylactic LVD may be recommended only for N1b PTMC with 3-level simultaneous metastasis.

## Author Contributions

All authors listed have made a substantial, direct and intellectual contribution to the work, and approved it for publication.

### Conflict of Interest Statement

The authors declare that the research was conducted in the absence of any commercial or financial relationships that could be construed as a potential conflict of interest.
