# Supplementary material for: Routine Lateral Level V Dissection May Not Be Necessary for Papillary Thyroid Microcarcinoma With Lateral Lymph Node Metastasis: A Retrospective Study of 252 Cases
Source: Front Endocrinol (Lausanne). 2019 Aug 20;10:558. doi: 10.3389/fendo.2019.00558 (PMC6710992; doi:10.3389/fendo.2019.00558)
Supplement: Supplementary file 1 [file Data_Sheet_1.doc]

**Supplementary Table 1: ROC analysis for optimal cutoff of continuous variables**

| Variables | AUC (95% CI) | P value | Youden Index | Cutoff |
| --- | --- | --- | --- | --- |
| LTD (cm) | 0.54(0.40-0.68) | 0.66 | 0.17 | >0.6 |
| Age | 0.56(0.42-0.69) | 0.52 | 0.22 | >32 |
| CLNM | 0.55(0.41-0.68) | 0.63 | 0.21 | ≤1 |

ROC: receiver operating characteristic curve; AUC: area under curve; LTD: largest tumor diameter; CLNM: central lymph node metastasis;
